# Social Motility in African Trypanosomes

**DOI:** 10.1371/journal.ppat.1000739

**Published:** 2010-01-29

**Authors:** Michael Oberholzer, Miguel A. Lopez, Bryce T. McLelland, Kent L. Hill

**Affiliations:** 1 Department of Microbiology, Immunology, and Molecular Genetics, University of California Los Angeles, Los Angeles, California, United States of America; 2 Molecular Biology Institute, University of California Los Angeles, Los Angeles, California, United States of America; Seattle Biomedical Research Institute, United States of America

## Abstract

African trypanosomes are devastating human and animal pathogens that cause significant human mortality and limit economic development in sub-Saharan Africa. Studies of trypanosome biology generally consider these protozoan parasites as individual cells in suspension cultures or in animal models of infection. Here we report that the procyclic form of the African trypanosome *Trypanosoma brucei* engages in social behavior when cultivated on semisolid agarose surfaces. This behavior is characterized by trypanosomes assembling into multicellular communities that engage in polarized migrations across the agarose surface and cooperate to divert their movements in response to external signals. These cooperative movements are flagellum-mediated, since they do not occur in trypanin knockdown parasites that lack normal flagellum motility. We term this behavior social motility based on features shared with social motility and other types of surface-induced social behavior in bacteria. Social motility represents a novel and unexpected aspect of trypanosome biology and offers new paradigms for considering host-parasite interactions.

## Author Summary

African trypanosomes, e.g. *Trypanosoma brucei*, and related kinetoplastid parasites cause morbidity and mortality in several million people worldwide. Trypanosomes are protists and are thus generally considered to behave as single-celled microorganisms. In other microorganisms, social interactions among individuals lead to development of multicellular communities with specialized and advantageous capabilities versus single cells. The concept of bacteria acting as groups of cells communicating and cooperating with one another has had a major impact on our understanding of bacterial physiology and pathogenesis, but this paradigm has not been applied to parasitic protozoa. Here we report that *T. brucei* is capable of social behavior when exposed to semisolid surfaces. This behavior, termed social motility, is characterized by the assembly of parasites into multicellular communities with emergent properties that are not evident in single cells. Parasites within communities exhibit polarized movements and cooperate to coordinate their movements in response to an external stimulus. Social motility offers many potential advantages, such as facilitating colonization and navigation through host tissues. The identification of social behavior in *T. brucei* reveals a novel and unexpected aspect of parasite biology and provides new concepts for considering host-parasite interactions.

## Introduction

Studying microbial life under conditions that promote a single-cell lifestyle has proven very effective for uncovering important aspects of microbial physiology. However, microbes are social organisms, capable of communicating with one another and engaging in cooperative behavior [1]–[3]. Well-characterized social activities include biofilm formation, social motility, fruiting body development and quorum sensing [1], [3]–[10]. Social interactions among cells in a population effectively give rise to multicellular communities having specialized functionalities and offering advantages over a unicellular lifestyle. Some of these advantages include increased protection from external antagonists, such as desiccation or host defenses, access to nutrients, exchange of genetic information and enhanced ability to colonize, penetrate and migrate on surfaces [1],[2],[11]. In bacterial and fungal pathogens, social interactions have major influences on microbial physiology and disease pathogenesis and considering multicellularity as a general property of bacteria has profoundly changed our understanding of microbiology [1]–[3],[6].

Most microorganisms, particularly pathogens, are intimately associated with surfaces in their natural environments and preferentially engage in social behavior when exposed to semisolid surfaces [2], [7], [10], [12]–[14]. Commonly, this is manifested as various forms of social motility, including swarming, gliding and twitching [12],[15]. Each of these surface-induced motilities is influenced by environmental and genetic factors and driven by overlapping yet distinct mechanisms that are not completely understood. The defining feature is cooperative movement of groups of bacteria across a surface, requiring active motility and cell-cell communication among members of the group in response to external stimuli. Once studied only in a few bacteria, such as *Proteus mirabilis* and *Serratia marcescens*, surface-induced cooperative motilities are now known to be widespread among both Gram-negative and Gram-positive bacteria, including several important pathogens, such as *Salmonella* and *Pseudomonas* spp. [13], [16]–[18]. Surface-induced social interactions have also been observed in yeasts and fungi, including the opportunistic pathogen *Candida albicans* [5],[6]. Thus, various types of surface-induced social behavior are widespread among microorganisms and applying this conceptual framework to studies of bacterial biology has yielded many novel insights. Surprisingly, the paradigm of social behavior has not previously been applied to parasitic protozoa.

African trypanosomes, i.e. *Trypanosoma brucei* and related species, are protozoan parasites that cause significant human mortality and limit economic development in sub-Saharan Africa [19]. *T. brucei* is transmitted to the bloodstream of a mammalian host through the bite of an infected tsetse fly vector. Parasite motility is important in both hosts and this is especially apparent in the tsetse, where parasites undergo an ordered series of directional migrations that are critical for parasite survival and completion of the life cycle [20]–[23]. Trypanosomes first colonize the midgut, then migrate into the ectoperitrophic space and advance back up the alimentary canal to the mouthparts and from there, to the salivary glands [21],[23]. Throughout this process, parasites are in intimate contact with tissue surfaces of the tsetse fly. Once in the salivary glands, epimastigotes colonize the epithelial surface, stimulating the final stage of differentiation into mammalian-infective trypomastigotes [22]–[24]. Thus, throughout the tsetse stage of its life cycle *T. brucei* is in intimate contact with host tissue surfaces and exhibits an implicit requirement for sensing and signaling to guide parasite migration and differentiation. Currently, little is known about how surface contact modulates trypanosome behavior or motility [25].

Here we report that *T. brucei* engages in social motility when cultivated on semisolid agarose surfaces. This behavior is characterized by the formation of multicellular communities that sense external stimuli and communicate with one another to coordinate movement of the population. *T. brucei* social motility shares features with surface-induced social behavior in other microorganisms and represents a novel form of motility and intercellular communication not previously observed in these pathogens. As such, our findings present a novel and unprecedented feature of trypanosome biology and provide new concepts for considering development and pathogenesis of parasitic protozoa.

## Results

**Figure 1:**
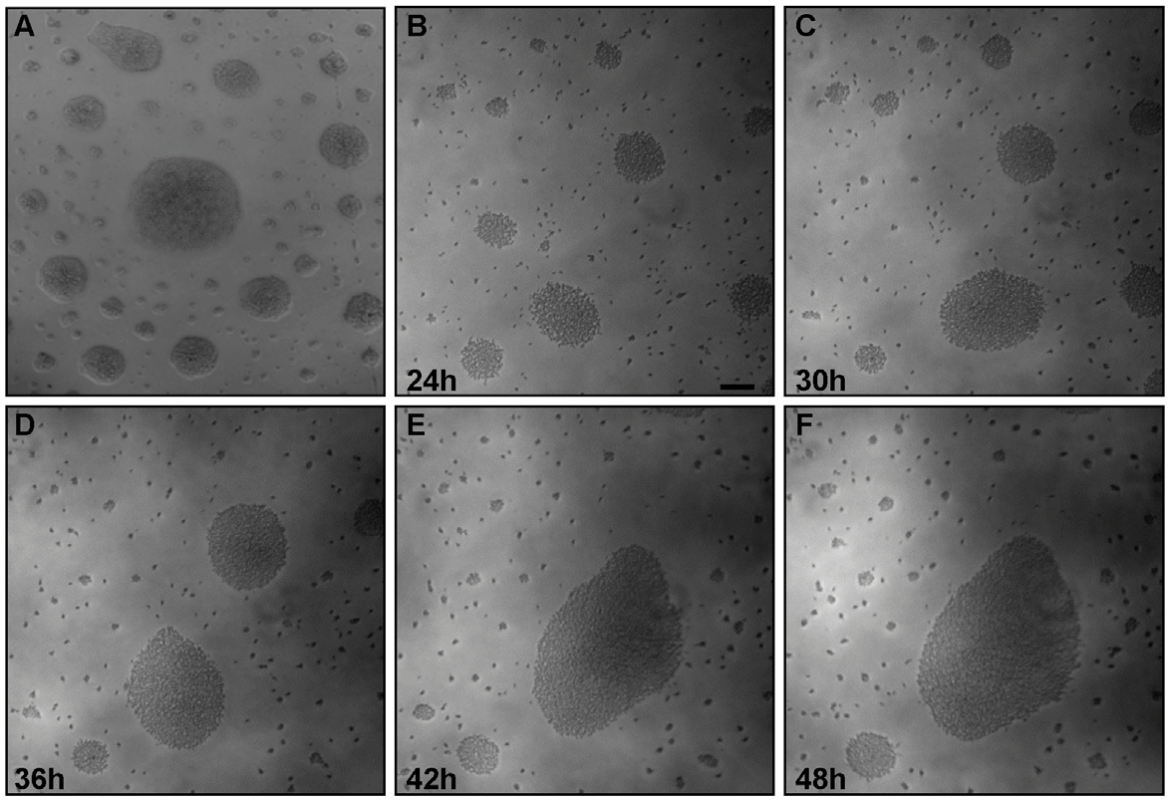
Trypanosome communities assemble through recruitment of neighboring cells. Trypanosome suspension cultures were transferred to semisolid agarose plates and monitored over time. (A) 48h post plating. (B–F) Time-lapse images show communities of parasites recruiting and merging with nearby individual cells and communities. The accompanying video (Video S1) shows groups of cells migrating en masse over the agarose surface. Scale bar is 100µm. Images are taken from Video S1 at the indicated time points post plating.

Surface-induced changes to microbial motility and behavior are common among diverse bacteria and protists [2],[5],[7],[26],[27]. *T. brucei* spends much of its life cycle in contact with host tissue surfaces and interaction between parasite and tsetse epithelia is well documented [22],[23],[28], yet studies of *T. brucei* motility to date mainly utilize suspension cultures and do not provide information about how parasite behavior, e.g. motility, is affected by contact with surfaces. As part of our ongoing investigations into trypanosome motility, we thus cultivated procyclic form *T. brucei* on semisolid agarose plates [29]. We focused on procyclic forms because we know more about the motility apparatus and have more mutants available in this life cycle stage than in bloodstream forms and because the potential impact of parasite motility is most pronounced in this stage [30]. We found that procyclic trypanosomes formed groups of densely-packed cells within 24h post-plating (Fig. 1). The approximate doubling time on plates was 24 hours (Fig. S3), indicating that these groups did not arise simply through clonal expansion of single cells. Individuals within each group remained highly motile and actively moved out and back from the group. Interestingly, parasites were often arranged in distinct patterns on the agarose surface, with large, tightly packed groups surrounded by a zone of clearance and then a perimeter of smaller groups (Fig. 1). To investigate how these patterns arose, we established a system to monitor parasite movements over several hours using time-lapse and video microscopy (Materials and Methods). Time-lapse imaging revealed a striking behavior in which groups of hundreds to thousands of parasites moved *en masse* across the agarose surface, recruiting neighboring cells and enabling mergers of large groups (Fig. 1B–F, Video S1). This confirmed that the assembly of large communities was an active process and not simply the result of clonal expansion.

**Figure 2:**
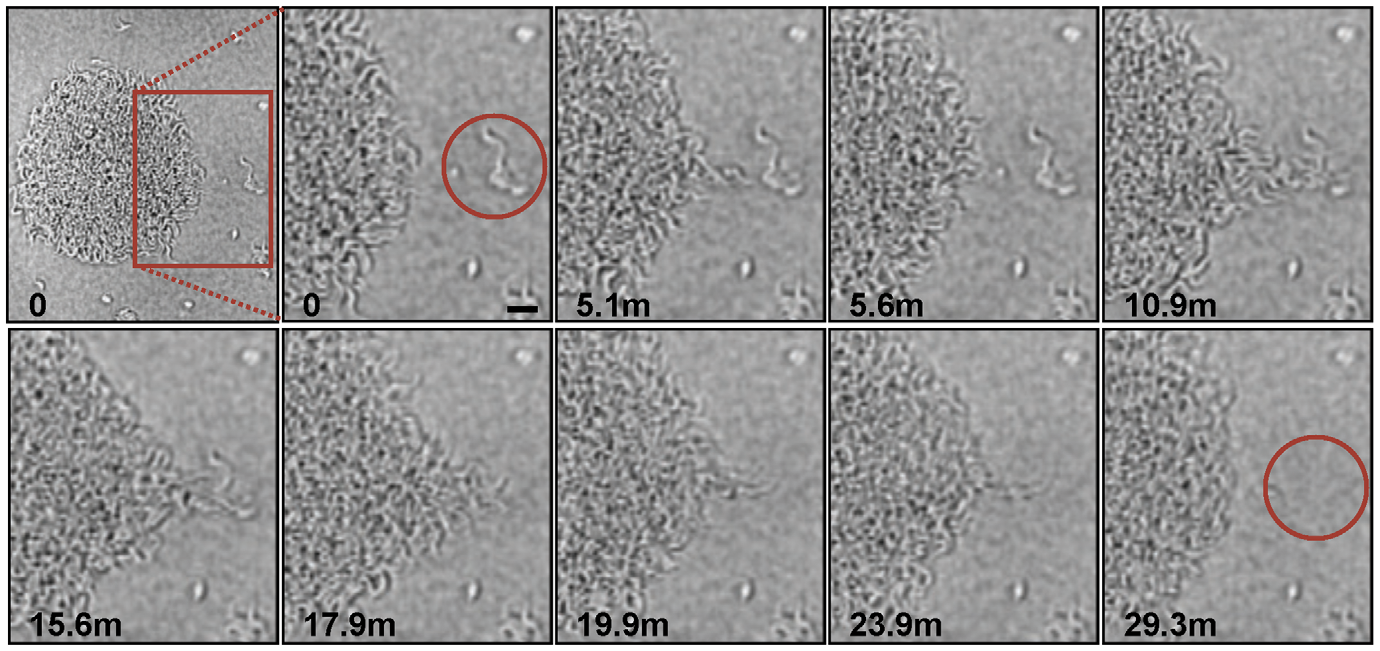
Parasite recruitment. Time-lapse images show active recruitment of individual cells into larger communities. Cells at the periphery of the community (termed “scouts”) move out and back. When these scouts come into contact with a group of cells outside the community and return (time points 5.1–5.6 min), they stimulate coordinated movement of cells at the community edge outward, toward the neighboring cells, leading to their recruitment into the community (time points 10.9–29.3min). The image series was initiated approximately 24 hours after plating and time points, in minutes, of individual images are indicated. Scale bar is 20µm. Images are taken from Video S2.

The *en masse* movement of large groups of trypanosomes across the agarose surface suggested some form of cooperation among individuals in the population. We therefore investigated this behavior more closely using increased magnification and greater time resolution (Fig. 2, Video S2). These analyses showed that recruitment of individual parasites into a community followed a specific sequence of events as described here. Cells at the periphery of the group were highly motile and moved out and back from the community. We refer to these cells as “scouts”. When scouts came into contact with cells located adjacent to the community, they returned and induced polarized movement of the community outward at this position, forming a multicellular “pseudopod” that extended to recruit the external parasites (Fig. 2, Video S2). Mergers of large groups of cells followed essentially the same sequence of events (Fig. S1, Video S3). First, single trypanosomes advanced and returned randomly from the group periphery. Second, contact of scouts with an adjacent group biased their movement and initiated a period of reciprocal exchange. This led to formation of a multicellular “pseudopod” between the groups that intermittently broke down and reformed. Ultimately, stable contact was made and the groups merged along a path defined by the “pseudopod”. Thus, the arrangement of cells on the agarose surface resulted from the cooperative movement of parasites into groups, which then expand through recruitment of neighboring cells.

**Figure 3:**
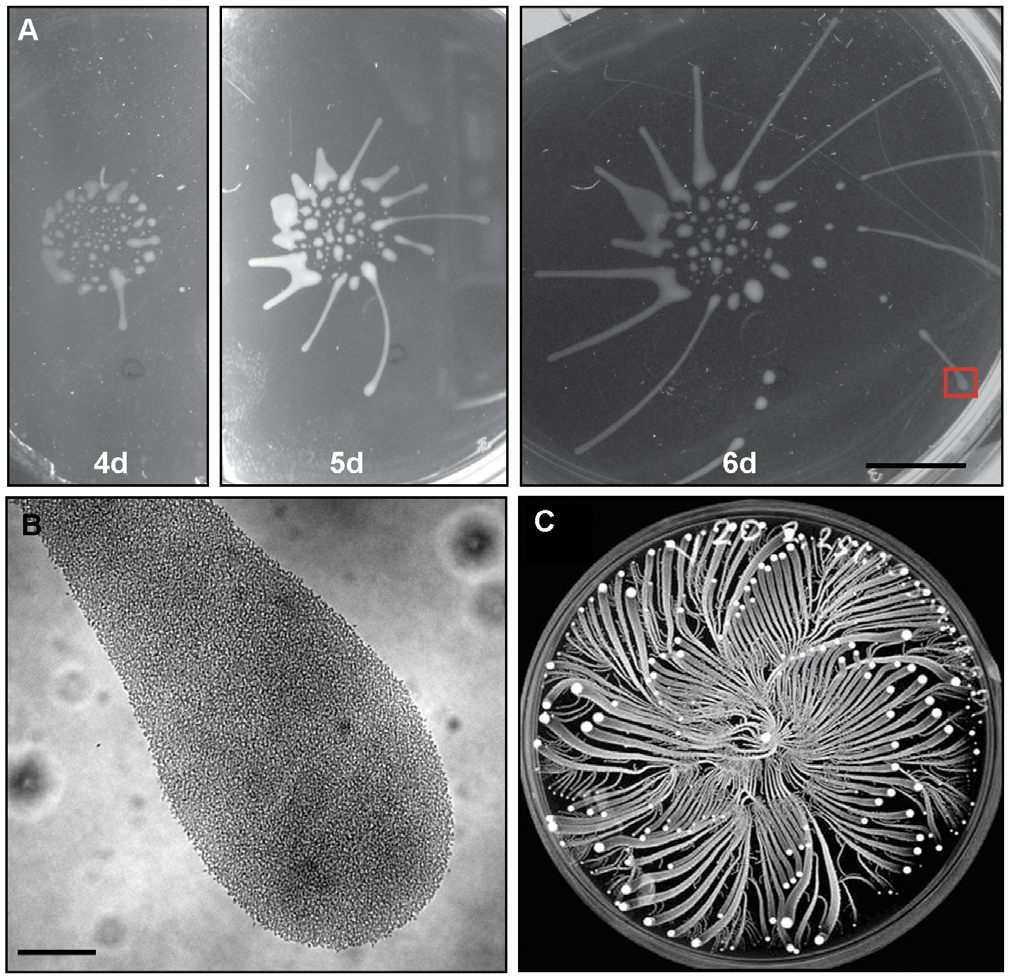
*T. brucei* social motility results in polarized migration outward from the site of inoculation. (A) Trypanosome communities 4 days (4d), 5 days (5d) and 6 days (6d) post plating. Parasites accumulate at the periphery of the inoculation site (4d, also compare to mot+ samples in Fig. 4A). Groups at the periphery then move outward, forming characteristic radial projections (5d and 6d). (B) Close up of the radial projection leading edge, (boxed region in panel A6d) shows characteristic bulbous accumulation of densely-packed cells. (C) Comparison to social motility patterns formed by *Paenibacillus vortex*, as shown by Ingham and coworkers [33]. Scale bars are 1cm (panel A), 200µm (panel B). Panel C adapted from [33], with permission.

Long-term cultivation of social bacteria on semisolid agarose gives rise to large macro-communities that form complex patterns on the agarose surface [2],[15],[17]. *T. brucei* formed macrocommunities within three to six days following inoculation (Fig. 3A). A characteristic feature of this process is that parasites initially collected into small clusters that were distributed around the perimeter of the inoculation site. Parasites in these clusters then advanced outward from the site of inoculation, forming symetrical arrays of radial projections, with a median of 13 projections per inoculum. This pattern is similar to that produced during social motility in *Pseudomonas aeruginosa*, *Myxococcus xanthus and Paenibacillus dendritiformis* [7], [13], [18], [31]–[33], as shown by others (Fig. 3C). Movement of trypanosome projections was polarized, with a single leading edge that advanced at a steady rate on the order of a few microns per minute (Fig. S2, Video S4). The leading edge was characterized by a bulbous accumulation of densely packed cells, while the proximal region maintained a constant width (Fig. 3B). Cells along the lateral edge readily moved out and back (Fig. S2, Video S5), demonstrating they are not physically restrained. Therefore, polarized migration of projections is governed by parasite actions, rather than physical restrictions on parasite movement.

**Figure 4:**
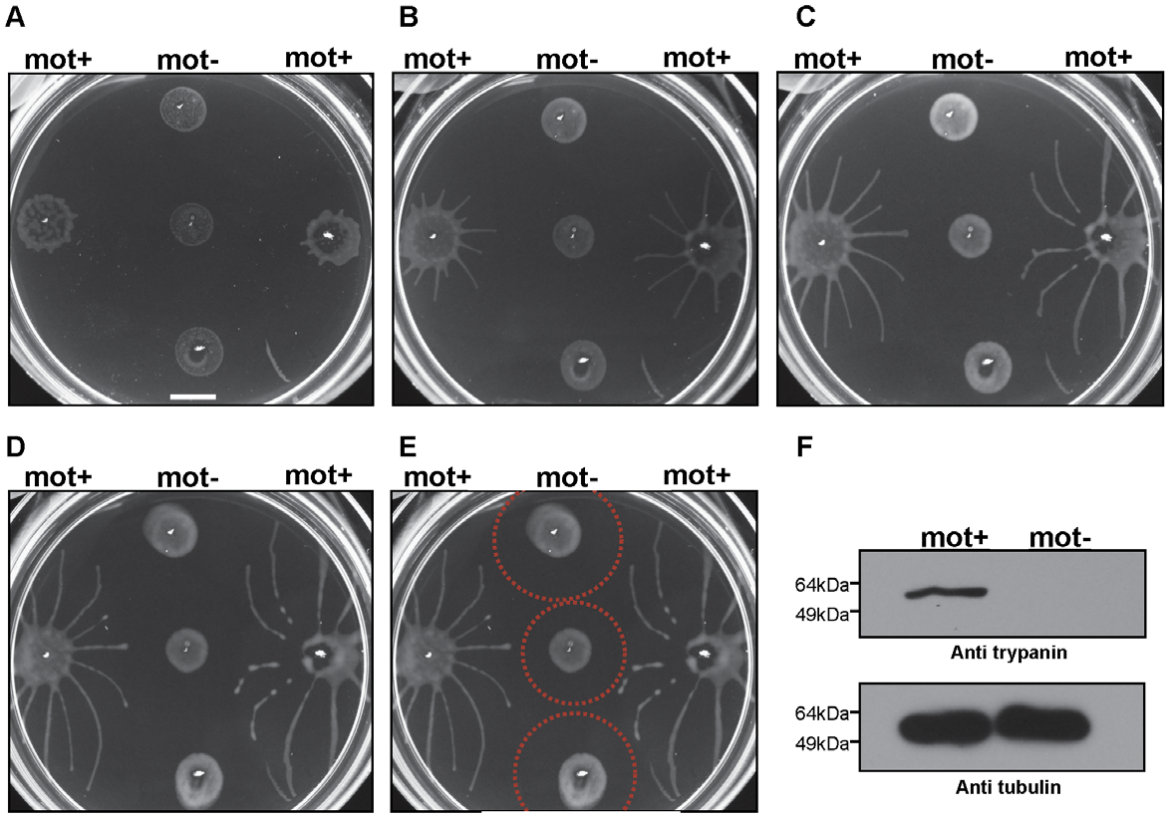
Social motility requires directional cell motility. Suspension cultures of control cells (mot +) or trypanin knockdown cells (mot −) were inoculated on semisolid agarose plates and monitored over time. (A–E) Images taken 3 (A), 4 (B), 5 (C), or 6 (D, E) days post plating. (E) Indicated by red circles is the approximate zone of inhibition. (F) Western blot analysis on proteins extracted from control (mot +) and trypanin knockdown (mot −) cells grown on the same agarose plate. Tubulin is used as a loading control. Scale bar in panel a is 1cm.

To determine whether social motility requires directional motility, we employed a trypanin RNAi knockdown line that is incapable of directional motility [34]. Trypanin knockdown cells were evenly distributed at the site of inoculation and did not accumulate at the perimeter, as seen for cells having wild type motility (Fig. 4A). Moreover trypanin knockdown cells did not form radial projections (Fig. 4A–D). Cell doubling continued normally, as indicated by the roughly equivalent increase in cell density over time versus control cells (Fig. S3). Trypanin knockdown and control cells from the same plate were collected and assayed for (i) cell number, (ii) RNAi induction and (iii) motility. Both groups demonstrated an approximately equal cell doubling (Fig. S3) and trypanin protein was undetectable in the knockdown cells (Fig. 4F). Absence of directional motility in trypanin knockdowns was confirmed by direct microscopic examination (data not shown). Therefore, social motility in trypanosomes requires directional motility and is an active process.

**Figure 5:**
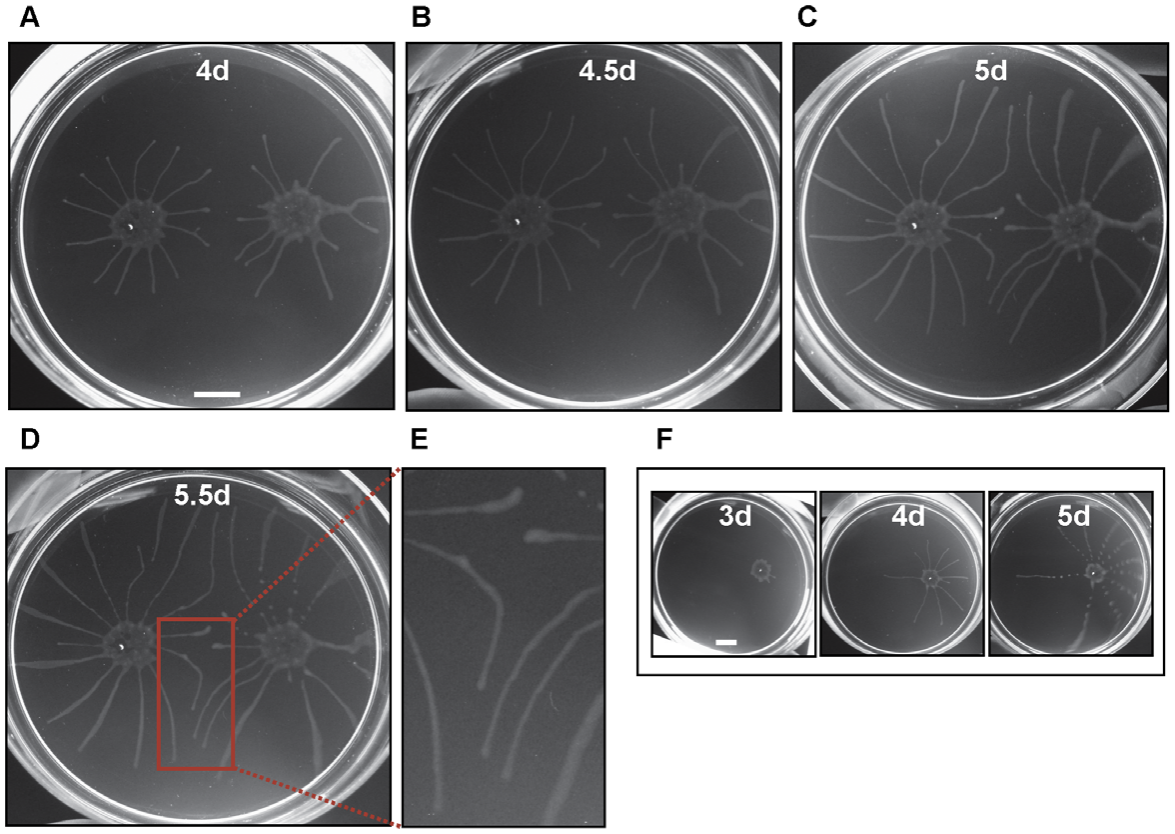
Trypanosomes sense nearby communities and change their course of migration. (A–D) Cells were inoculated on opposing sides of the agarose plate and monitored at the number of days post plating indicated in each panel. Projections radiating from each inoculation site advance outward in a generally straight path unless they come in proximity with opposing projections, at which point they halt progression, or redirect their movement. (E) Close up of outlined area in panel D. (F) As a control, cells from a single inoculation advance outward in a generally linear path to the edge of the culture dish. Scale bars are 1cm.

Radial projections advanced in parallel and did not cross paths (Fig. 4A–D). Moreover, when projections from a control group approached a non-motile group, their movement was either halted or was diverted so as to avoid contact (Fig. 4A–D). When diverted, projections did not cross, rather they continued in parallel, implying that cells in each projection are capable of sensing each other and coordinating their movements. Avoidance of non-motile communities occurred within a radius of approximately 0.5–1 cm (Fig. 4E). To determine if this avoidance was uniquely a response to non-motile cells, we inoculated two groups of control cells on opposing sides of a culture plate and followed their development and migration over the course of several days (Fig. 5). Opposing radial projections either halted advancement, or diverted paths so as to avoid contact with one another. As a negative control, radial projections from a single community of motile cells did not divert their path of migration over time (Fig. 5F). The combined data thus indicate that *T. brucei* can sense and respond to external signals and that parasites in a community can sense other parasites and may choose to include them in the group or to avoid them.

## Discussion

The impact of cell-cell communication and a multicellular lifestyle on the physiology and pathogenesis of bacteria is now well-established and related phenomena operate in yeast and fungi [1],[3],[5],[6],[35]. To date however, the paradigm of microbial social interactions has not been applied to parasitic protozoa. We report here that *T. brucei* is capable of social behavior in which parasites communicate with one another and assemble into multicellular communities with emergent properties that are not evident in single cells. This behavior manifests as groups of parasites engaging in cooperative movement across the surface of semisolid agarose and altering course in response to an external stimulus. We term this behavior social motility, based on analogy to social motility in bacteria. These results demonstrate a novel feature of trypanosome biology and reveal a level of complexity and cooperativity to trypanosome behavior that was not previously recognized. Given the widespread distribution of social interactions among other microbes, we expect our findings to have broad relevance among parasitic protozoa.

Social interactions among microbes are manifested in a variety of forms and represent complex behavioral responses for which the underlying molecular mechanisms are not well-understood. As is the case for bacteria [2], social motility in *T. brucei* requires directional motility, involves some form of cellular differentiation upon exposure to a semisolid surface and culminates in cooperative cell migration in response to external signals. At early stages parasites merge into groups, while at later stages the behavior has the added feature of groups avoiding one another. This suggests some form of differentiation and is consistent with different stages of social motility observed in some bacteria, such as *Paenibacillus* spp. [33]. In most cases where it has been investigated, social motility requires a combination of external and internal, i.e. genetic, factors and it is likely that this is also the case in trypanosomes. Based on our observations and what is known in other organisms, a minimum requirement for social motility in *T. brucei* would be directional motility, the ability to sense an external signal and to transduce this signal into a cellular response and communication between parasites in a group. Trypanosomes are certainly capable of directional motility [36],[37] and must integrate host-derived and parasite-derived signals to complete their life cycle [24], [38]–[40], although their signaling and sensory capacities are poorly understood. The trypanosome genome encodes several components of classical signal transduction pathways, as well as numerous predicted cell surface proteins of unknown function that might serve sensory and/or signaling roles [40]–[45]. The contribution of these proteins to cell-cell signaling or other sensory functions is not known and efforts to address this question have been limited by the lack of a defined in vitro assay for cell-cell signaling. Social motility assays therefore provide an opportunity to test the requirement of trypanosome signaling systems in social motility and overcome a major barrier to dissecting signaling and sensory mechanisms in trypanosomes.

Within the tsetse, close contact between parasites, as well as intimate interactions with host tissue surfaces are readily observed [20],[22],[23], indicating that surface-induced social behavior might operate in vivo. However, until appropriate mutants are available for direct investigation, we can only speculate on potential physiological roles for social motility. In this context it is informative to consider whether there are features of the parasite life cycle that might benefit from social motility or related behavior. *T. brucei* development within the tsetse fly requires parasite migration across and through a variety of host tissues. These migrations lead ultimately to colonization of the tsetse salivary gland epithelia, which the parasites must reach in order to complete development into mammalian-infective trypomastigotes. Trypanosomes progress through specific tsetse tissues in a well-defined order, but the mechanisms responsible for tissue tropisms are unknown. Social motility offers a system in which groups of cells coordinate their movements in response to an external stimulus and thus could provide a mechanism for parasite navigation through host tissues. In bacterial pathogens, cell-cell signaling, assembly into multicellular communities, social motility and other types of surface-induced behavior provide several advantages. Groups of bacteria feed cooperatively, resist hostile environments, prey on other microbes, exchange genetic information and develop functional specializations [1],[2]. Quorum sensing and biofilm formation induce programs of virulence gene expression, facilitate colonization of host tissues and provide resistance to immune and physical defenses [3]. We speculate that trypanosome cell-cell communication and social behavior may have similar impacts on development and pathogenesis of *T. brucei*. For example, assembly into groups might facilitate resistance against host defenses in the tsetse [24], as well as promote tissue colonization and invasion. Social motility might also provide a means to bring parasites together for genetic exchange [46],[47]. Finally, signaling pathways required for social motility are expected to overlap with host-parasite signaling pathways, about which very little is known. In summary, the identification of social motility in *T. brucei* reveals a novel and unexpected aspect of parasite biology and provides entirely new conceptual approaches for considering host-parasite interactions.

## Materials and Methods

### Trypanosome cell lines and suspension culture

Three procyclic *T. brucei brucei* cell lines, Antar 1 R5 Pro/G ITM [21], 29-13 double marker [48], and trypanin RNAi (KHTb12) [34], were used for these studies. While each experiment was not duplicated for each cell line, social motility was observed for both Antar and 29-13 lines. Suspension cultures were maintained using Cunningham's semi-defined medium (SM), supplemented with 10% heat-inactivated fetal calf serum as described previously [49]. For 29-13 cells, the medium was further supplemented with 15µg/ml G418 (Gibco) and 50µg/ml Hygromycin (Gibco). For the trypanin RNAi line, 2.5µg/ml Phleomycin, 15µg/ml G418 (Gibco) and 50µg/ml Hygromycin (Gibco) were included in the medium and RNAi was induced by adding 1µg/ml tetracycline. Cell doubling was monitored using a Z1 Coulter Particle Counter (Beckman Coulter, USA).

### Plating on semisolid agarose plates

Cultivation on semi-solid agarose plates was adapted from [29]. Four percent (w/v) agarose (SeaPlaque GTG Agarose, Cambrex-LONZA, ME, USA) solution was made in MiliQ water, autoclaved and cooled to 65°C. A 1∶10 dilution of this 4% stock solution was prepared in pre-warmed (42°C for 20min) SM culture medium supplemented with the appropriate antibiotics for selection. The resulting 0.4% agarose solution was cooled to 37°C for 1h. In most cases ethanol (final concentration 1%) was added to the medium. A 13ml aliquot was poured into Petri Dishes (100×15mm), which were then dried without lids for 1.5h in a laminar flow hood at room temperature. For inoculation onto the plate, 5µl of cells from a suspension culture at a density of 1.5×10^7^cells/ml were added on the agarose surface. For the experiments in Fig. 2 and S1, 50ul of cells were spread on the surface by gently rotating and rocking the plate. Trypanin RNAi lines were induced for 72h with 1µg/ml tetracycline in suspension culture prior to plating. Inoculated plates were dried for 3 min without lids, closed and sealed with parafilm and incubated as for suspension cultures at 27°C.

### Imaging of plates

For Fig. 1A, the plate was imaged using a Zeiss Axioskop II microscope with a 2.5×LD Plan NeoFluor objective and Zeiss Axiocam camera. For Fig. 1B–F (Video S1), the plate was imaged using a Zeiss Axiovert 200M microscope with a 2.5×LD Plan NeoFluor objective and a COHU RS-170 high performance CCD camera (COHU, Inc.). Images were captured at 1 frame per 10min at room temperature using Adobe premiere Elements 1.0 (Adobe Systems). Time stamps are indicated in the panels. For Video S1, images were compiled into a movie using NIH-ImageJ (http://rsbweb.nih.gov/ij). The playback speed is 5 frames per second (3000× original speed) and elapsed time is 24h.

For Fig. 2 (Video S2) and Fig. S1 (Video S3), plates were maintained at 28°C, 5% CO_2_ in a CTI humidified live cell cultivation chamber equipped with heating insert and CTI 3700 controller from Zeiss, Inc. This chamber allows independent control of humidity, temperature and CO_2_ on the microscope stage. Plates were monitored on a Zeiss Axiovert 200M microscope, using a 10×LD Plan NeoFluor objective and a COHU RS-170 High performance CCD camera (COHU, Inc.). For Fig. 2 (Video S2), images were captured once every 5 sec and played back at 10 frames per second (fps), giving a final playback speed of 50×. For Fig. S1 (Video S3), the video was recorded in real-time using a VCR, then digitized in AVI format at 30 frames per second (fps) using an in-line Sony Handycam digital camera as an analog/digital converter and Adobe Premier Elements (Adobe Systems). Individual images were extracted at 1 fps, exported into QuickTime video format using the Sorenson TM CODEC within Adobe Premier Elements, and played back at 30 fps. The final playback speed is thus 30× real speed and elapsed time is 10 minutes 57 seconds.

For Fig. 3A, 4A–E and 5, plates were imaged at the indicated times post plating using an Olympus Stylus 770 SW digital camera and processed using Adobe Photoshop 8.0. For Fig. 3B, the plate was imaged as described above for Fig. 1B–F.

For Fig. S2 (Videos S4 and S5), time-lapse images were captured and compiled into video as described above for Fig. 1B–F. The playback speed is 6429× and elapsed time is 21.43 hours for Video S4 and 8.9h for Video S5.

### Western blotting

Cells were collected in PBS from the agarose plate, counted and washed two times in PBS. The equivalent increase in opaqueness of control and trypanin RNAi communities on plates, Fig. 4, indicated that they continued doubling at equivalent rates and direct cell counting confirmed this. Protein samples were prepared and subjected to Western blot analysis as described [49], using 1×10^6^ cell equivalents per lane. Monoclonal anti-trypanin antibody [50] was used at 1∶5000, and monoclonal anti-β-tubulin E7 hybridoma supernatant was used at 1∶5000. The anti-β-tubulin antibody was developed by Michael Klymkowsky, University of Colorado and was obtained from the Developmental Studies hybridoma Bank developed under the auspices of the NICHD and maintained by the University of Iowa, Department of Biological Sciences, Iowa City, IA 52242. Secondary antibody was horseradish peroxidase-coupled goat anti-mouse (BioRad) used at 1∶2500.

## Supporting Information

Figure S1 Merger of large communities occurs via the same defined sequence of events that drive recruitment of individual cells. High frame-rate (30 frames/sec) video analysis demonstrates that groups of parasites detect the presence of adjacent groups and then merge. Mergers occur in discrete stages defined as follows. Cells migrate out and back from each group giving an undulating appearance to the group periphery (A). Contact with an adjacent group initiates a period of reciprocal exchange (B–F), followed by stable contact, then rapid and massive cell movement as the two groups merge (G–H). Cell movement between groups generates multicellular pseudopod-like projections (black arrow). These projections only form between adjacent groups and only after contact. Therefore, crosstalk between groups by just a few cells initiates directed and coordinated movement of the entire group. Essentially the same sequence of events drives recruitment of individual cells into a group (Fig. 2). Scale bar is 20µm. Time-lapse image series taken from Video S3.
(1.94 MB TIF)

Figure S2 Radial projections advance exclusively at the leading edge, even though cells at the lateral edge are free to move out and back. (A, B) Snapshots of the leading edge (*) of a migrating community at time point 0 (A), and 21.3 hours later (B). Projections advanced at a steady rate of 2.3µm/min, as determined from this movie (Video S4). Scale bar 1cm. (C) Close-up of the region boxed in B. Cells at the lateral edge freely move in and out (black arrows). Scale bar is 1 cm. Image taken from Video S5.
(1.88 MB TIF)

Figure S3 Cell doubling of control and trypanin RNAi strains on semi solid agarose. Plates were inoculated with the same number (6.5×10^4^ cells) of 29-13 control (mot+) or trypanin RNAi (mot −) cells. Cells from each community were collected by rinsing with PBS at the indicated number of days (3d, 4d and 5d) post inoculation and counted using a hemacytometer (A). At each time-point, the plates were imaged (B) prior to harvesting cells. The data show averages and standard deviations calculated from four communities for each time-point for each cell line. Scale bar in panel B is 1cm.
(1.48 MB TIF)

Video S1 Trypanosome communities assemble through recruitment of neighboring cells. This movie corresponds to the time-lapse images in Fig. 1B–F and shows merger of cells and communities of cells. The elapsed time is 24 hr.
(1.85 MB MOV)

Video S2 Parasite recruitment. Video S2 corresponds to the time-lapse images in Fig. 2 and shows high magnification of a trypanosome community recruiting neighboring cells. The elapsed time is 29.3 minutes.
(3.83 MB MOV)

Video S3 Merger of large communities occurs via the same defined sequence of events that drive recruitment of individual cells. This movie corresponds to the time-lapse images in Fig. S1 and shows specific stages of merger between trypanosome communities. The elapsed time is 10 minutes, 57 seconds.
(3.56 MB AVI)

Video S4 Merger of large communities occurs via the same defined sequence of events that drive recruitment of individual cells. This movie corresponds to the time-lapse images in Fig. S1 and shows specific stages of merger between trypanosome communities. The elapsed time is 10 minutes, 57 seconds.
(10.39 MB MOV)

Video S5 Cells at the lateral edge of radial projections are free to move out and back. This movie corresponds to the image in Fig. S2C and shows that cells along the lateral edge of advancing radial projections freely move out and back. The elapsed time is 8.9 hours.
(0.84 MB MOV)
