# Supplementary figures and images for: Social Motility in African Trypanosomes
Source: PLoS Pathog. 2010 Jan 29;6(1):e1000739. doi: 10.1371/journal.ppat.1000739 (PMC2813273; doi:10.1371/journal.ppat.1000739)

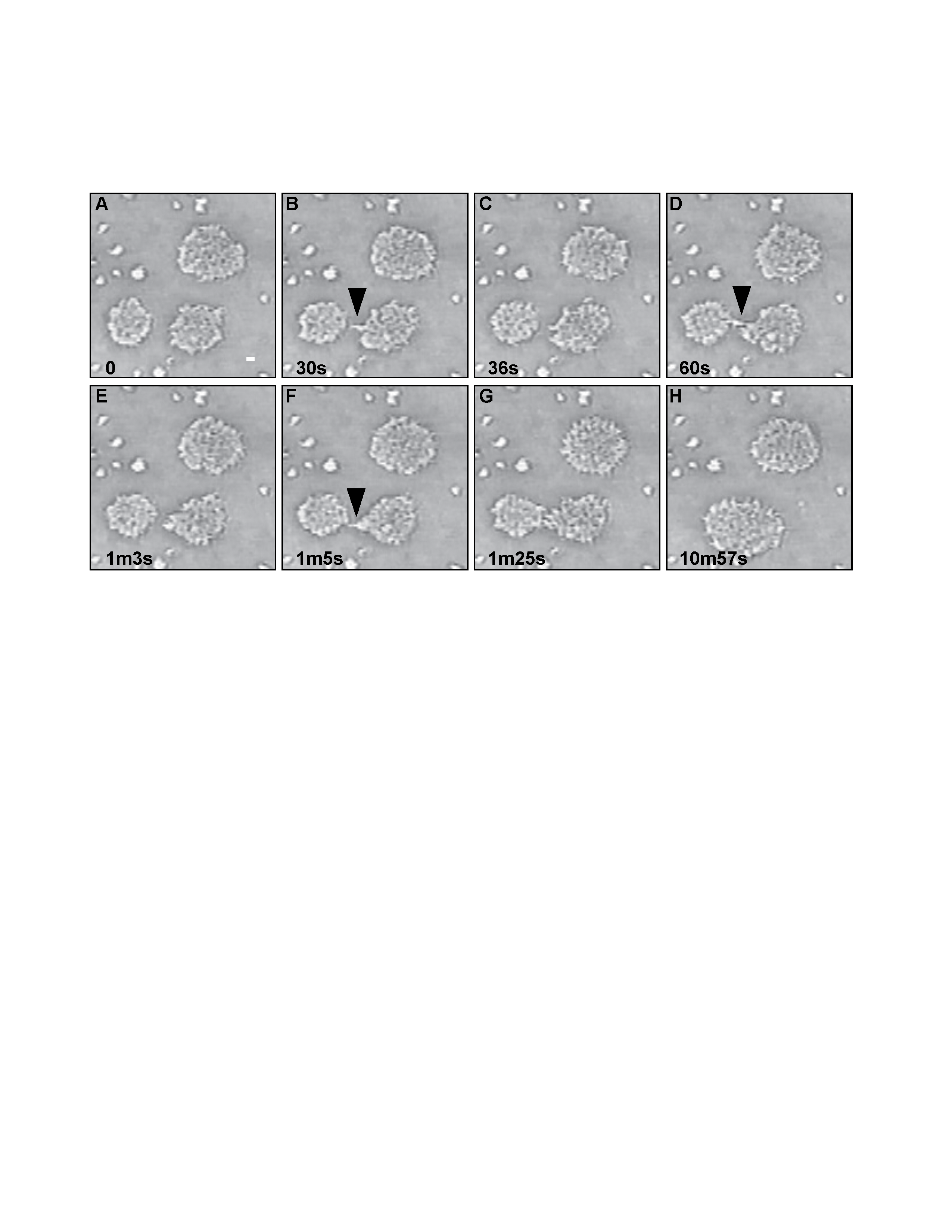

Supplement: Figure S1 — Merger of large communities occurs via the same defined sequence of events that drive recruitment of individual cells. High frame-rate (30 frames/sec) video analysis demonstrates that groups of parasites detect the presence of adjacent groups and then merge. Mergers occur in discrete stages defined as follows. Cells migrate out and back from each group giving an undulating appearance to the group periphery (A). Contact with an adjacent group initiates a period of reciprocal exchange (B–F), followed by stable contact, then rapid and massive cell movement as the two groups merge (G–H). Cell movement between groups generates multicellular pseudopod-like projections (black arrow). These projections only form between adjacent groups and only after contact. Therefore, crosstalk between groups by just a few cells initiates directed and coordinated movement of the entire group. Essentially the same sequence of events drives recruitment of individual cells into a group (Fig. 2). Scale bar is 20µm. Time-lapse image series taken from Video S3. (1.94 MB TIF) [file ppat.1000739.s001.tif]

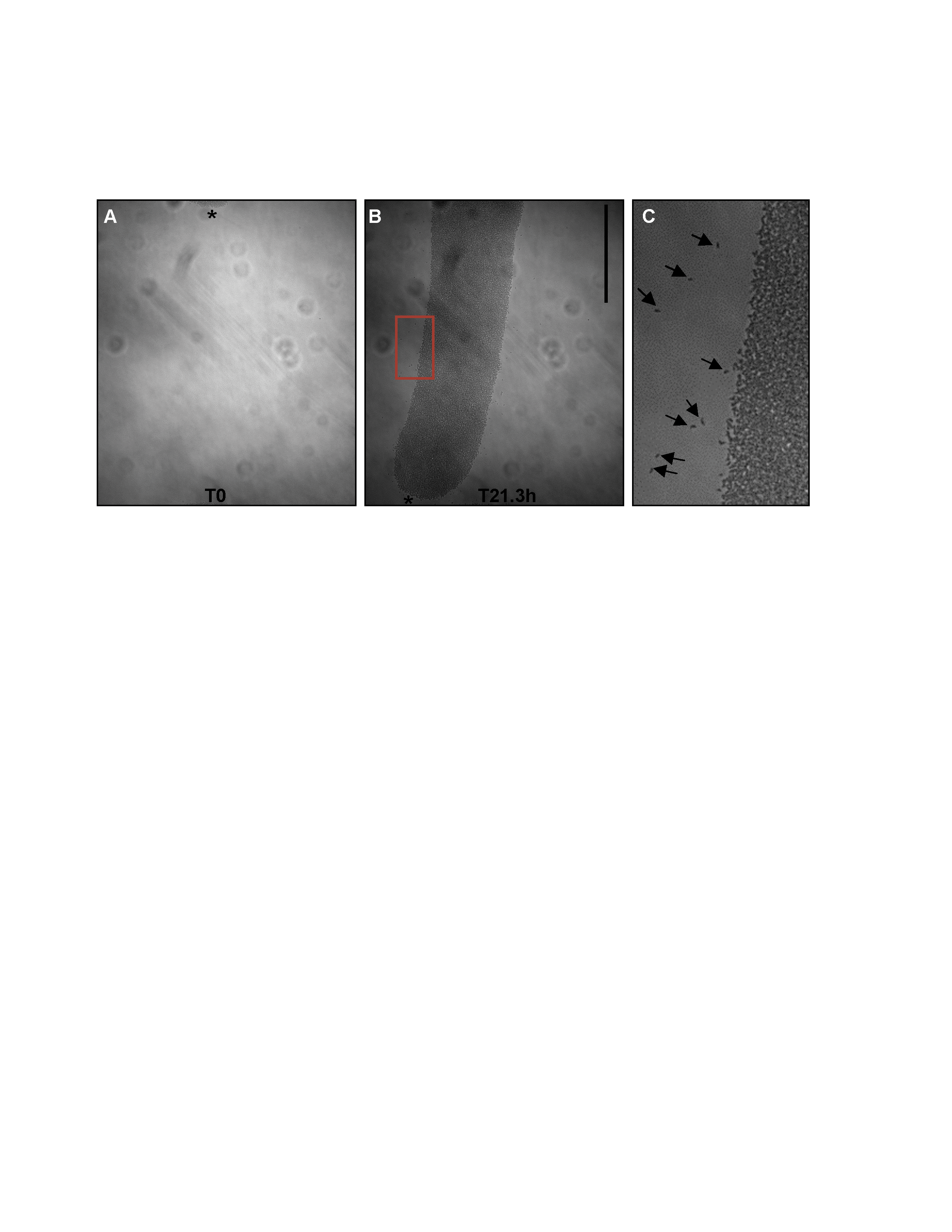

Supplement: Figure S2 — Radial projections advance exclusively at the leading edge, even though cells at the lateral edge are free to move out and back. (A, B) Snapshots of the leading edge (*) of a migrating community at time point 0 (A), and 21.3 hours later (B). Projections advanced at a steady rate of 2.3µm/min, as determined from this movie (Video S4). Scale bar 1cm. (C) Close-up of the region boxed in B. Cells at the lateral edge freely move in and out (black arrows). Scale bar is 1 cm. Image taken from Video S5. (1.88 MB TIF) [file ppat.1000739.s002.tif]

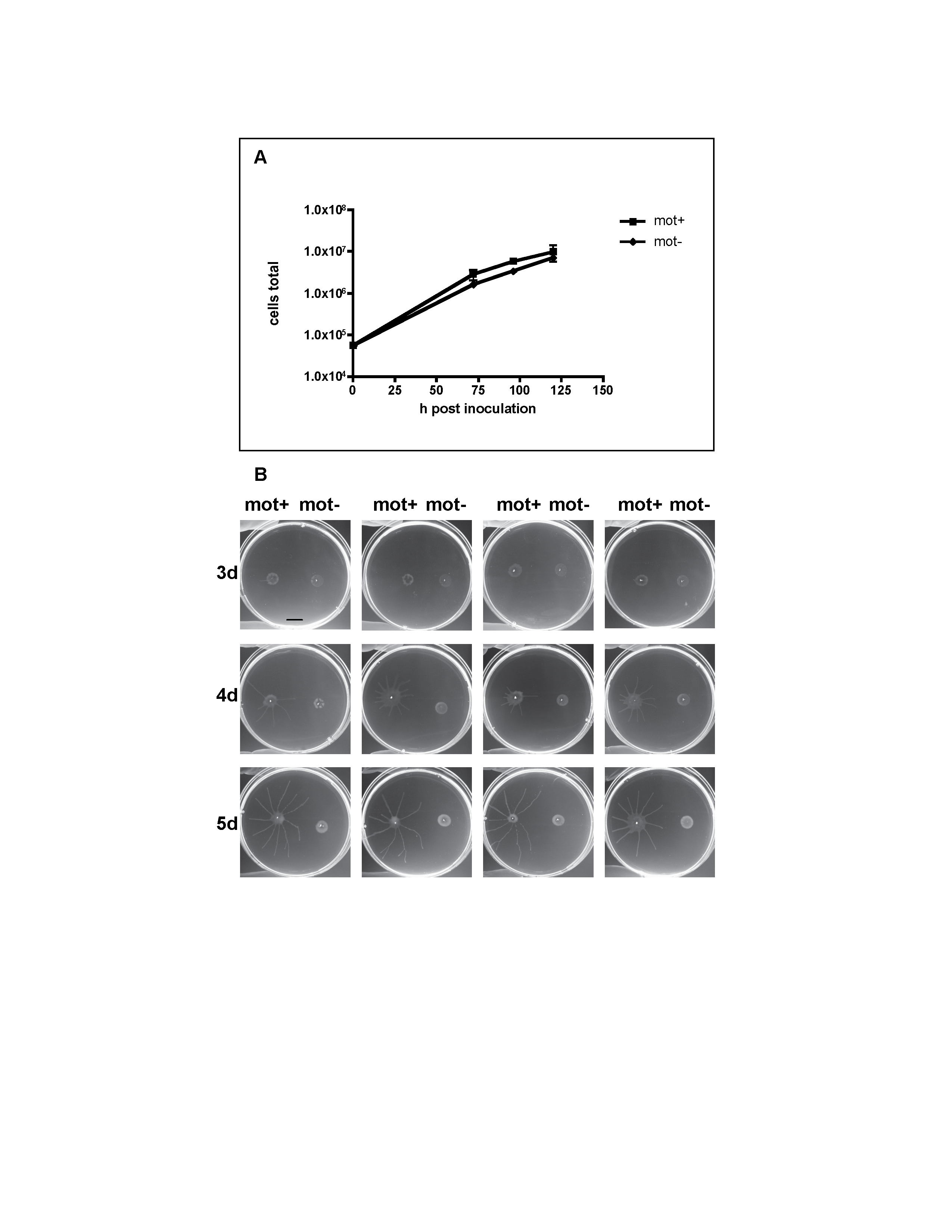

Supplement: Figure S3 — Cell doubling of control and trypanin RNAi strains on semi solid agarose. Plates were inoculated with the same number (6.5×10^4^ cells) of 29-13 control (mot+) or trypanin RNAi (mot −) cells. Cells from each community were collected by rinsing with PBS at the indicated number of days (3d, 4d and 5d) post inoculation and counted using a hemacytometer (A). At each time-point, the plates were imaged (B) prior to harvesting cells. The data show averages and standard deviations calculated from four communities for each time-point for each cell line. Scale bar in panel B is 1cm. (1.48 MB TIF) [file ppat.1000739.s003.tif]
